# Association Between the Atherogenic Index of Plasma and the Occurrence of Slow Flow/No‐Reflow in Patients With Acute Myocardial Infarction Undergoing Emergency Percutaneous Coronary Intervention

**DOI:** 10.1155/crp/4736031

**Published:** 2026-09-12

**Authors:** Qinyu Sun, Yifan Deng, Weiyi Zhou, Zhoujun Huang, Shenghu He, Jing Zhang, Wei Zhou

**Affiliations:** ^1^ Department of Cardiology, Northern Jiangsu People’s Hospital Affiliated to Yangzhou University, Yangzhou 225001, Jiangsu, China, yzu.edu.cn; ^2^ Department of Cardiology, Northern Jiangsu People’s Hospital, Yangzhou 225001, Jiangsu, China, yzsbh.com; ^3^ Department of Rheumatology and Immunology, Affiliated Hospital of Yangzhou University, Yangzhou 225000, Jiangsu, China, yzu.edu.cn

**Keywords:** acute myocardial infarction, percutaneous coronary intervention, slow flow/no-reflow, the atherogenic index of plasma

## Abstract

**Background:**

Percutaneous coronary intervention (PCI) is the primary reperfusion strategy for acute myocardial infarction (AMI); however, some patients develop slow flow/no‐reflow (SF/NR) after the procedure, which adversely affects prognosis. Atherogenic index of plasma (AIP) is a reliable biomarker of cardiovascular risk, yet its predictive value for SF/NR following emergency PCI in AMI patients remains unclear.

**Objective:**

This study aimed to investigate the association between AIP and the occurrence of SF/NR in AMI patients undergoing emergency PCI.

**Methods:**

A retrospective analysis was conducted on 964 patients initially diagnosed with AMI who underwent PCI. The participants were categorized into SF/NR (*n* = 251) and normal flow (*n* = 713) groups on the basis of TIMI flow grade. Statistical analyses including LASSO regression, multivariate logistic regression, ROC curve analysis, RCS regression, subgroup analysis, and the development of a nomogram model were performed.

**Results:**

AIP was identified as an independent risk factor for postoperative SF/NR (OR = 3.184, *p* < 0.05). The AUC for AIP in the prediction of SF/NR was 0.737. Higher AIP quartiles were associated with increased risk, and a nonlinear relationship was observed. The constructed nomogram model demonstrated good predictive performance (AUC = 0.745), satisfactory calibration in internal validation, and favorable clinical utility. Subgroup analysis confirmed the consistent predictive ability of AIP across different strata.

**Conclusion:**

AIP may be associated with the risk of SF/NR after emergency PCI in AMI patients. Combined with white blood cell count and triglyceride levels, AIP could potentially assist in early identification of high‐risk individuals, which warrants further validation in prospective studies.

**Trial Registration:** Chinese Clinical Trial Registry: ChiCTR2500108501

## 1. Introduction

Cardiovascular diseases, particularly acute myocardial infarction (AMI), represent a major global health challenge [1]. Percutaneous coronary intervention (PCI) is the preferred reperfusion strategy for AMI and is widely used in clinical practice. This technique rapidly restores blood flow in the infarct‐related artery, significantly improves myocardial perfusion, effectively alleviates symptoms, and enhances clinical outcomes [2]. However, some patients may still experience inadequate perfusion at the myocardial tissue level after successful revascularization—a phenomenon known as slow flow/no‐reflow (SF/NR)—which is among the key factors associated with adverse outcomes after PCI [3]. Therefore, the rapid and accurate identification of SF/NR in AMI patients following PCI is critically important for clinical prognosis.

The atherogenic index of plasma (AIP) is a composite lipid parameter calculated as the logarithmically transformed ratio of triglycerides (TGs) to high‐density lipoprotein cholesterol (HDL‐C). It has been established in multiple studies as a reliable biomarker for various conditions, including dyslipidemia [4], diabetes [5], and stroke [6]. It has been reported that AIP predicts slow coronary flow in patients with chronic coronary syndrome [7]. However, it remains unclear whether the AIP can predict the occurrence of SF/NR following PCI in patients with AMI. This study aimed to investigate the association between the AIP and the incidence of SF/NR after PCI in AMI patients.

## 2. Methods and Subjects

### 2.1. Study Population and Grouping

**FIGURE 1:**
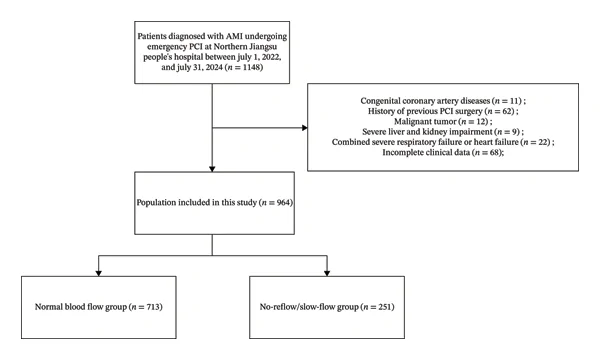
Patient inclusion flowchart.

A retrospective analysis was conducted on patients initially diagnosed with AMI who underwent PCI at Northern Jiangsu People’s Hospital between July 2022 and July 2024 (Figure 1). Among the enrolled patients, 617 (64.0%) were diagnosed with ST‐segment elevation myocardial infarction (STEMI) and 347 (36.0%) with non‐ST‐segment elevation myocardial infarction (NSTEMI). Patients were classified into two groups on the basis of the thrombolysis in myocardial infarction (TIMI) flow grade: those with SF/NR (TIMI grades 0–2, *n* = 251) and those with normal reflow (TIMI grade 3, *n* = 713), after mechanical causes such as coronary artery spasm or occlusion were excluded. The categorization of TIMI grade 2 as “slow flow” rather than “no‐reflow” was based on established literature [8, 9].

Inclusion criteria are as follows:

1. Age between 18 and 90 years;
2. Meeting the diagnostic criteria for AMI [10];
3. Availability of complete clinical data.

Exclusion criteria are as follows:

1. The regular use of anticoagulant or antiplatelet agents for the secondary prevention of coronary heart disease (CHD);
2. Preadmission use of lipid‐modifying medications such as statins or fibrates;
3. Nonobstructive causes of AMI including coronary artery bridging or coronary artery spasm;
4. Severe hepatic or renal dysfunction;
5. Severe heart failure, cardiogenic shock, other organic heart diseases (e.g., valvular heart disease, malignant arrhythmias), or a history of cardiac surgery;
6. Comorbidities such as hyperthyroidism, severe infection, malignancy, immune system disorders, or hematologic diseases.

This study was approved by the Ethics Committee of Northern Jiangsu People’s Hospital (approval no. 2025ky251). The requirement for informed consent was waived because of the retrospective nature of the study.

### 2.2. Clinical Data Collection and AIP Calculation

General patient information—including age, smoking history, alcohol use, history of Type 2 diabetes, and history of hypertension—was collected through the electronic medical record system.

Hypertension was defined as a systolic blood pressure ≥ 140 mm Hg and/or diastolic blood pressure ≥ 90 mm Hg measured on three separate occasions.

Type 2 diabetes was defined as a previous confirmed diagnosis, current use of glucose‐lowering agents, fasting blood glucose ≥ 7.0 mmol/L, or 2‐h postprandial blood glucose ≥ 11.1 mmol/L.

Smoking was defined as the consumption of more than one cigarette per day for more than 6 months.

Heavy alcohol use was defined as an intake exceeding 50 mL per occasion, more than three times per week, for at least six consecutive months.

Venous blood samples were collected promptly after admission to measure routine parameters such as hemoglobin and white blood cell count. Following a 12‐h fast, additional venous blood was drawn the next morning to assess fasting blood glucose and lipid profiles. The left ventricular ejection fraction was accurately evaluated via biplane Simpson’s method.

AIP was calculated via the following formula: AIP = log[TG (mmol/L)/HDL‐C (mmol/L)].

### 2.3. PCI Procedure

All enrolled patients received standard antiplatelet pretreatment before PCI, consisting of 300 mg of aspirin and 300 mg of clopidogrel (or 180 mg of ticagrelor). Coronary angiography was performed in strict accordance with established protocols. Image analysis was conducted independently by two interventional cardiologists, who meticulously interpreted the results to assess the degree of coronary artery stenosis accurately. The culprit vessel was identified on the basis of comprehensive evaluation, including symptoms such as chest pain or chest tightness, electrocardiographic findings, and cardiac necrosis markers. Emergency PCI was subsequently performed on the culprit vessel. Post‐PCI coronary blood flow was evaluated using the TIMI flow grade. Poststent dilation was performed at the operator’s discretion based on lesion characteristics and stent expansion adequacy. Patients who developed SF/NR were typically treated clinically with adenosine, calcium channel blockers, sodium nitroprusside, nitroglycerine, or a combination thereof.

### 2.4. Statistical Analysis

Statistical analyses were performed via SPSS 27.0 and R 4.4.1. The normality of continuous variables was assessed via the Kolmogorov–Smirnov test. Normally distributed data are expressed as the mean ± standard deviation (*X* ± *S*) and were compared via the *t* test; nonnormally distributed data are expressed as the median (P_25_, P_75_) and were analyzed via the Mann–Whitney U test. Categorical variables are presented as percentages and were compared via the chi‐square test. Least absolute shrinkage and selection operator (Lasso) regression and multivariate logistic regression were employed to identify independent risk factors for SF/NR after emergency PCI in AMI patients. The AIP was analyzed both as a continuous variable and in quartiles, with the results reported as odds ratios (ORs) and 95% confidence intervals (CIs). The predictive performance of the AIP for SF/NR was evaluated via receiver operating characteristic (ROC) curve analysis. The potential nonlinear association between AIP and SF/NR was examined via restricted cubic spline (RCS) regression. A nomogram prediction model was constructed on the basis of the regression results. A multicollinearity analysis was performed on the indicators used to construct the model, and all variance inflation factors were below 5, and tolerances were above 0.2, indicating no significant multicollinearity. Model validation included (1) calibration via the Hosmer–Lemeshow test and internal validation via the bootstrap resampling method (1000 repetitions); (2) discrimination evaluated via ROC analysis; and (3) clinical utility assessed via decision curve analysis (DCA). Subgroup analyses were conducted on the basis of smoking status, alcohol use, hypertension, and diabetes to explore potential interactions between these factors and the association of the AIP with SF/NR.

## 3. Results

### 3.1. Comparison of the General Characteristics Between the Two Groups

A total of 964 patients were included in this study, comprising 693 males and 271 females, with a median age of 64 (50, 77) years. Compared with the normal flow group, the SF/NR group presented significantly greater values for the following parameters: male sex, smoking status, atrial fibrillation, hemoglobin, white blood cell count, neutrophil count, monocyte count, platelet count, fasting blood glucose, glycosylated hemoglobin A1c, TGs, apolipoprotein B, and AIP (all *p* < 0.05). Conversely, HDL‐C and apolipoprotein A1 were significantly lower in the SF/NR group (all *p* < 0.05). No statistically significant differences were observed in the remaining indicators (all *p* ≥ 0.05), as detailed in Table 1.

**TABLE 1:** Comparison of the general characteristics between the two groups.

Variables	Total (*n* = 964)	Normal reflow (*n* = 713)	Slow flow/no‐reflow (*n* = 251)	Statistic	*p*
Gender [*n*, (%)]				*χ* ^2^ = 4.902	0.027
Female	271 (28.112)	214 (30.014)	57 (22.709)		
Male	693 (71.888)	499 (69.986)	194 (77.291)		
Smoking [*n*, (%)]				*χ* ^2^ = 6.212	0.013
No	480 (49.793)	372 (52.174)	108 (43.028)		
Yes	484 (50.207)	341 (47.826)	143 (56.972)		
Drinking [*n*, (%)]				*χ* ^2^ = 1.619	0.199
No	708 (73.444)	516 (72.370)	192 (76.494)		
Yes	256 (26.556)	197 (27.630)	59 (23.506)		
Hypertension [*n*, (%)]				*χ* ^2^ = 2.340	0.126
No	272 (28.216)	192 (26.928)	80 (31.873)		
Yes	692 (71.784)	521 (73.073)	171 (68.127)		
Diabetes [*n*, (%)]				*χ* ^2^ = 2.305	0.129
No	652 (67.635)	492 (69.004)	160 (63.745)		
Yes	312 (32.365)	221 (30.996)	91 (36.255)		
Atrial fibrillation [*n*, (%)]				*χ* ^2^ = 7.642	0.006
No	956 (99.170)	711 (99.719)	245 (97.610)		
Yes	8 (0.830)	2 (0.281)	6 (2.390)		
Number of diseased vessels [*n*, (%)]				*χ* ^2^ = 2.675	0.262
1	435 (45.124)	319 (44.741)	116 (46.215)		
2	446 (46.266)	338 (47.405)	108 (43.028)		
3	83 (8.610)	56 (7.854)	27 (10.757)		
Age [M (P_25_, P_75_), year]	64 (50, 77)	64 (50, 77)	64 (49, 77)	*Z* = −0.742	0.458
BMI [M (P_25_, P_75_), kg/m^2^]	26.354 (24.096, 28.656)	26.491 (24.107, 28.665)	26.082 (24.043, 28.637)	*Z* = −0.507	0.612
LVEF [M (Q_1_, Q_3_), (%)]	56 (45, 62)	56 (44, 62)	55 (45, 61)	*Z* = −0.697	0.486
Hemoglobin [M (P_25_, P_75_), g/L]	130.000 (113.000, 142.000)	129.000 (112.000, 141.000)	132.000 (116.000, 144.000)	*Z* = −2.220	0.026
White blood cells [M (P_25_, P_75_), × 10^9^/L]	5.005 (1.718, 7.647)	4.240 (1.690, 6.911)	6.870 (1.940, 9.280)	*Z* = −6.033	< 0.001
Neutrophil count [M (P_25_,P_75_),× 10^9^/L]	3.090 (0.520, 5.340)	2.530 (0.490, 4.630)	4.470 (0.800, 7.120)	*Z* = −6.993	< 0.001
Lymphocyte count[M (P_25_, P_75_),× 10^9^/L]	1.740 (1.308, 2.152)	1.750 (1.320, 2.165)	1.720 (1.235, 2.105)	*Z* = −0.374	0.709
Monocyte count [M (P_25_, P_75_), × 10^9^/L]	0.440 (0.320, 0.560)	0.420 (0.310, 0.520)	0.500 (0.380, 0.720)	*Z* = −6.259	< 0.001
Platelet count [M (P_25_, P_75_), × 10^9^/L]	127.000 (44.900, 198.000)	104.000 (44.400, 189.000)	171.000 (47.050, 222.000)	*Z* = −5.072	< 0.001
Fasting blood glucose [M (P_25_, P_75_), mmol/L]	5.270 (4.350, 6.081)	5.150 (4.270, 5.920)	5.509 (4.710, 6.854)	*Z* = −3.808	< 0.001
Glycosylated hemoglobin, Type A1C [M (P_25_, P_75_), %]	5.700 (4.478, 6.600)	5.600 (4.240, 6.424)	5.900 (5.100, 6.950)	*Z* = −4.605	< 0.001
Triglycerides [M(P_25_, P_75_), mmol/L]	1.220 (0.960, 1.600)	1.150 (0.940, 1.470)	1.510 (1.100, 2.085)	*Z* = −8.527	< 0.001
Total cholesterol [M (P_25_, P_75_), mmol/L]	4.650 (3.960, 5.332)	4.660 (4.000, 5.310)	4.580 (3.870, 5.390)	*Z* = −0.646	0.519
High‐density lipoprotein cholesterol [M (P_25_, P_75_), mmol/L]	1.460 (1.040, 1.580)	1.480 (1.070, 1.600)	1.240 (0.950, 1.530)	*Z* = −4.790	< 0.001
Low‐density lipoprotein cholesterol [M (P_25_, P_75_), mmol/L]	1.810 (1.280, 2.900)	1.820 (1.280, 2.990)	1.760 (1.300, 2.715)	*Z* = −1.027	0.305
Lipoprotein a [M(P_25_, P_75_), mg/L]	68.350 (0.980, 208.525)	70.900 (0.980, 229.100)	61.700 (1.000, 184.250)	*Z* = −0.785	0.433
Apolipoprotein A1 [M (P_25_, P_75_), g/L]	1.590 (1.260, 41.100)	1.710 (1.280, 41.200)	1.450 (1.185, 40.350)	*Z* = −2.405	0.016
Apolipoprotein B [M (P_25_, P_75_), g/L]	1.280 (0.890, 24.712)	1.310 (0.930, 25.000)	1.170 (0.820, 22.762)	*Z* = −2.483	0.013
Albumin [M (P_25_, P_75_), g/L]	40.213 (32.200, 45.625)	40.500 (32.200, 46.000)	39.810 (32.500, 44.500)	*Z* = −1.141	0.254
ALT [M (P_25_, P_75_), U/L]	56.000 (25.000, 205.000)	57.980 (25.000, 207.000)	48.000 (25.150, 191.750)	*Z* = −1.033	0.302
AST [M (P_25_, P_75_), U/L]	66.000 (30.000, 90.438)	66.000 (30.000, 90.250)	66.000 (31.500, 90.537)	*Z* = −0.312	0.755
Potassium [M (P_25_, P_75_), mmol/L]	3.840 (3.610, 4.130)	3.840 (3.620, 4.130)	3.840 (3.580, 4.120)	*Z* = −0.615	0.539
Uric acid [M (P_25_, P_75_), μmol/L]	240.550 (79.000, 349.028)	232.800 (78.200, 346.000)	259.000 (81.500, 352.888)	*Z* = −1.202	0.229
Creatinine [M (P_25_, P_75_), μmol/L]	78.000 (61.000, 99.000)	77.900 (61.000, 98.500)	81.000 (60.250, 99.050)	*Z* = −0.343	0.732
Hs‐CRP [M (P_25_, P_75_), mg/L]	18.265 (6.000, 29.000)	18.430 (7.000, 28.749)	18.000 (4.150, 29.654)	*Z* = −0.445	0.657
AIP [M (P_25_, P_75_)]	−0.024 (−0.140, 0.109)	−0.047 (−0.182, 0.033)	0.090 (−0.020, 0.223)	*Z* = −11.175	< 0.001

### 3.2. Lasso and Multivariate Logistic Regression Analysis

**FIGURE 2:**
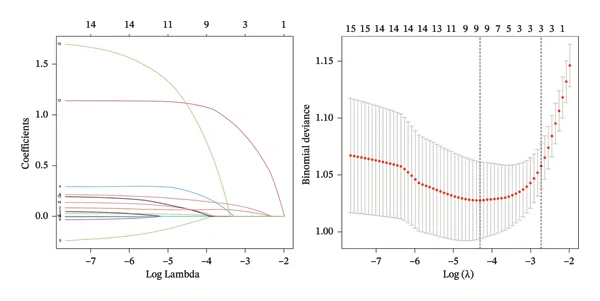
Lasso regression analysis.

To mitigate multicollinearity, variables showing significant differences in univariate analysis—including male sex, smoking, atrial fibrillation, hemoglobin, white blood cell count, neutrophil count, monocyte count, platelet count, fasting blood glucose, glycosylated hemoglobin A1c, TGs, HDL‐C, apolipoprotein A1, apolipoprotein B, and AIP—were included in the LASSO regression analysis. This process identified nine relevant factors: male sex, smoking, atrial fibrillation, white blood cell count, neutrophil count, monocyte count, TGs, HDL‐C, and AIP (Figure 2).

These nine factors were subsequently entered into a multivariate logistic regression model via a backward stepwise selection method. The analysis revealed three independent risk factors for SF/NR: white blood cell count (OR = 1.114, 95% CI: 1.067–1.163), TGs (OR = 1.218, 95% CI: 1.031–1.440), and AIP (OR = 3.184, 95% CI: 2.162–4.689) (Table 2).

**TABLE 2:** Multivariate logistic regression analysis.

Variables	β	S.E	*Z*	*p*	OR (95%CI)
White blood cells	0.108	0.022	4.891	< 0.001	1.114 (1.067–1.163)
Triglycerides	0.197	0.085	2.319	0.020	1.218 (1.031–1.440)
AIP	1.158	0.197	5.866	< 0.001	3.184 (2.162–4.689)

For all indicators ultimately included in the model, we assessed multicollinearity. The results showed that white blood cell count (VIF = 1.124 and tolerance = 0.890), TGs (VIF = 1.924 and tolerance = 0.520), and AIP (VIF = 1.835 and tolerance = 0.545) had variance inflation factors below 5 and tolerance values above 0.2, indicating no significant multicollinearity among these variables.

### 3.3. Predictive Value of the AIP for SF/NR After Emergency PCI in AMI Patients

**FIGURE 3:**
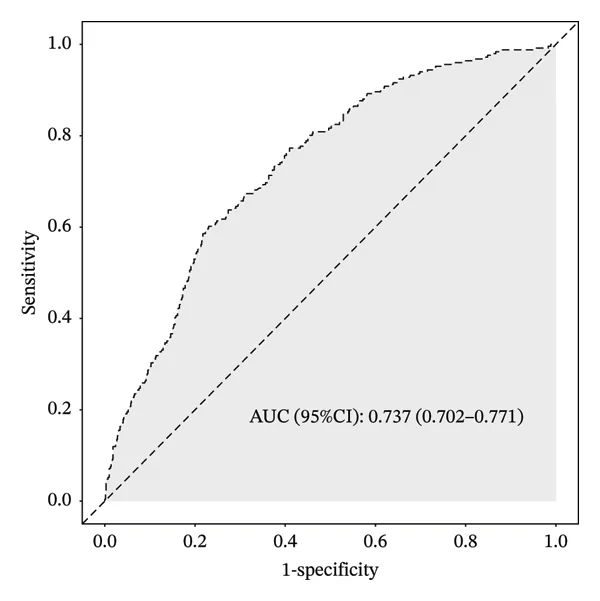
ROC curve for AIP in predicting slow flow/no reperfusion after emergency PCI in AMI patients.

ROC curve analysis demonstrated that the AIP effectively predicted the occurrence of SF/NR following emergency PCI in patients with AMI, with an AUC of 0.737 (95% CI: 0.702–0.771). The sensitivity and specificity were 77.0% and 60.2%, respectively (Figure 3).

### 3.4. Multivariate‐Model Logistic Regression Analysis of AIP

When analyzed as a continuous variable, the AIP demonstrated a statistically significant association with SF/NR across all three models (*p* < 0.05). When analyzed as a categorical variable (quartiles), patients were divided into the following groups: Q1 (*n* = 244, AIP ≤ −0.140), Q2 (*n* = 235, −0.140 < AIP ≤ −0.024), Q3 (*n* = 91, −0.024 < AIP ≤ 0.109), and Q4 (*n* = 394, AIP > 0.109). The risk of SF/NR significantly increased with increasing AIP quartile (*p* < 0.05, Table 3).

**TABLE 3:** Multivariate‐model logistic regression analysis of AIP.

Variables	Model 1		Model 2		Model 3		Model 4	
	OR (95% CI)	*p*	OR (95% CI)	*p*	OR (95% CI)	*p*	OR (95% CI)	*p*
AIP	4.719 (3.400–6.550)	< 0.001	4.200 (3.008–5.865)	< 0.001	3.630 (2.547–5.173)	< 0.001	3.604 (2.523–5.148)	< 0.001
Q1	—		—		—		—	
Q2	2.822 (1.553–5.127)	< 0.001	2.655 (1.454–4.849)	< 0.001	2.657 (1.461–4.834)	0.001	2.579 (1.412–4.711)	< 0.001
Q3	4.783 (2.427–9.427)	< 0.001	4.171 (2.091–8.318)	< 0.001	4.372 (2.213–8.640)	< 0.001	4.011 (2.011–8.001)	< 0.001
Q4	10.030 (5.893–17.068)	< 0.001	8.562 (5.003–14.652)	< 0.001	7.623 (4.360–13.327)	< 0.001	7.445 (4.251–13.041)	< 0.001

*Note:* Model 1: crude. Model 2: adjust: white blood cells. Model 3: adjust: triglycerides. Model 4: adjust: white blood cells, triglycerides.

Abbreviations: CI, confidence interval; OR, odds ratio.

To address concerns regarding mathematical redundancy between AIP and its components, we excluded TGs from the multivariate model. In this Model 3, AIP remained a significant independent predictor (*p* < 0.001), and the direction and magnitude of the association were similar to the primary analysis.

### 3.5. RCS Analysis of the Association Between AIP and SF/NR After Emergency PCI in AMI Patients

**FIGURE 4:**
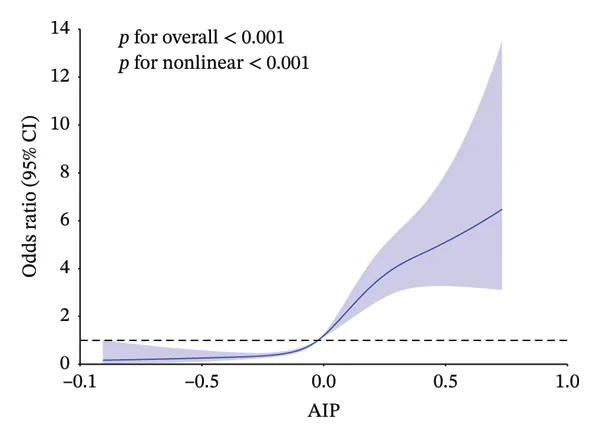
RCS curves for slow flow/no reperfusion events following emergency PCI in AIP and AMI patients.

RCS analysis, adjusted in Model 3, revealed that the overall association between the AIP and the risk of SF/NR was statistically significant (*p* overall < 0.001), with a notable nonlinear component (*p* nonlinear < 0.001). As the AIP increased, the risk of the outcome rose significantly, demonstrating a distinct nonlinear dose‒response relationship (Figure 4).

### 3.6. Development and Evaluation of the Predictive Model

**FIGURE 5:**
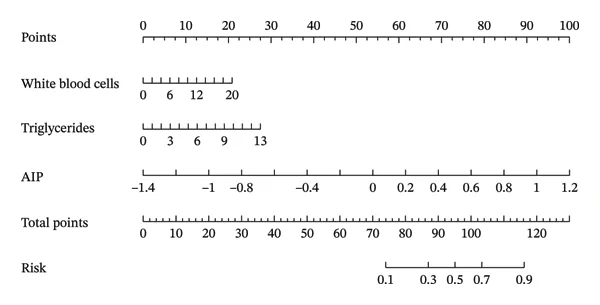
Column chart of the prediction model.

**FIGURE 6:**
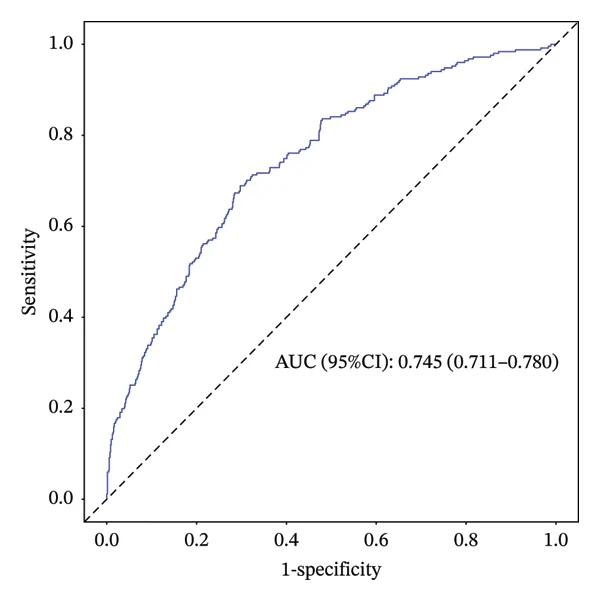
ROC curve of the predictive model.

On the basis of the results of the multivariate logistic regression analysis, a nomogram model was constructed to predict the risk of SF/NR after emergency PCI in AMI patients by incorporating the white blood cell count, TG level, and AIP (Figure 5). The area under the ROC curve for the nomogram was 0.745 (95% CI: 0.711–0.789), indicating good discriminative ability (Figure 6). Internal validation was performed via the bootstrap resampling method with 1000 repetitions (*B* = 1000). The calibration curve showed a mean absolute error of *p* = 0.812 (> 0.05, *x* ^2^ = 6.92, df = 8), with no significant difference between predicted and observed probabilities. (Supporting Information Figure 1). DCA demonstrated a favorable net benefit across a reasonable threshold probability range, supporting the clinical utility of the nomogram (Supporting Information Figure 2).

### 3.7. Subgroup Analysis

To further investigate the association between the AIP and the occurrence of SF/NR after emergency PCI in AMI patients, subgroup analyses were performed (Table 4). The results indicated that the AIP consistently demonstrated predictive value across various subgroups stratified by smoking history, alcohol consumption, hypertension, and diabetes. A significant positive correlation between the AIP and the risk of SF/NR was observed in all subgroups (all interaction *p* values > 0.05), suggesting that the association remained stable regardless of these potential influencing factors.

**TABLE 4:** Subgroup analysis.

Variables	*n* (%)	OR (95% CI)	*p* for interaction
All patients	964 (100.00)	4.72 (3.40–6.55)	
Smoking			0.340
No	480 (49.79)	3.93 (2.47–6.27)	
Yes	484 (50.21)	5.42 (3.40–8.63)	
Drinking			0.142
No	708 (73.44)	4.12 (2.85–5.95)	
Yes	256 (26.56)	7.64 (3.65–15.97)	
Hypertension			0.737
No	272 (28.22)	4.38 (2.46–7.80)	
Yes	692 (71.78)	4.94 (3.31–7.38)	
Diabetes			0.139
No	652 (67.63)	3.95 (2.67–5.84)	
Yes	312 (32.37)	6.85 (3.70–12.66)	

## 4. Discussion

The SF/NR phenomenon is a serious complication that may occur after successful revascularization of the infarct‐related artery and is closely associated with adverse patient outcomes. Therefore, active prevention and management of this phenomenon are crucial for improving the clinical prognosis. Studies have shown that the incidence of no‐reflow can reach as high as 32% in patients with acute coronary syndrome (ACS) [11]. Currently, the TIMI flow grade criterion is the most widely used and clinically valuable tool for assessing coronary blood flow perfusion [12]. In this study, SF/NR flow was defined as a TIMI flow grade of 0–2 after PCI. The results indicated that 251 patients (26.03%) developed this complication. This threshold aligns with previous investigations that included TIMI 2 flow as a marker of incomplete myocardial reperfusion [8, 9, 13], although we acknowledge that TIMI 2 flow is not universally equated with “no‐reflow” and that a finer distinction between TIMI 0–1 and TIMI 2 might have yielded additional insight. Nevertheless, the 26.03% incidence observed in our cohort is consistent with the range of 5%–32% reported in the literature [13]. Early identification of patients at high risk of SF/NR, enhanced perioperative monitoring, and optimization of preoperative preparation and interventional strategies are highly important for minimizing its occurrence and potential harm.

The multivariable logistic regression analysis performed in this study revealed that the AIP was an independent risk factor for SF/NR after emergency PCI in patients with AMI (OR = 3.184, 95% CI: 2.162–4.689). When analyzed as a continuous variable, the AIP showed a clear dose‒response relationship with the incidence of SF/NR, an association that remained consistent when the AIP was categorized into quartiles (Q1–Q4). ROC curve analysis confirmed the predictive value of the AIP, with an area under the curve (AUC) of 0.737 (95% CI: 0.702–0.771), a sensitivity of 77.0%, and a specificity of 60.2%. Both subgroup analysis and RCS regression demonstrated a robust association between AIP and the risk of SF/NR. This positive correlation remained stable across all subgroups after adjusting for multiple potential confounding factors.

While previous studies have reported an association between AIP and no‐reflow in STEMI and ACS cohorts [14, 15], the present study extends these findings in several important ways. First, our cohort includes both STEMI and NSTEMI patients, reflecting an AMI population. Second, we performed a detailed nonlinear analysis using RCS regression, revealing a distinct dose–response pattern that has not been previously characterized. Third, we constructed and internally validated a nomogram integrating AIP with white blood cell count and TGs, offering a practical bedside tool for risk stratification. These features contribute novel evidence to the existing literature by providing a more comprehensive assessment of the AIP–SF/NR relationship across the entire spectrum of AMI.

The AIP was proposed by Shi and Wen [16] as a means to estimate the level of small, dense low‐density lipoprotein cholesterol (sdLDL‐C). When calculated as the logarithm of the ratio of TG to HDL‐C, the AIP inversely correlates with the particle size of sdLDL‐C and serves as a simple yet effective marker for sdLDL‐C content. sdLDL‐C is defined as low‐density lipoprotein particles with an average diameter smaller than 25.5 nm. Owing to their distinct physicochemical properties, these particles exhibit increased atherogenic potential [17]. They more readily penetrate the arterial intima, have lower affinity for the LDL receptor, remain in the bloodstream for a longer duration, and are more susceptible to oxidation and modification. These characteristics promote lipid uptake by macrophages and the formation of foam cells, thereby accelerating the progression of atherosclerosis [18] and potentially increasing the risk of SF/NR events. Studies have shown that the AIP may offer superior predictive value for cardiovascular and cerebrovascular risk compared with conventional lipid parameters such as HDL‐C, LDL‐C, total cholesterol (TC), or TG alone [19]. Thus, elevated AIP may serve as an effective population‐level screening tool to identify individuals at high risk of developing SF/NR after PCI.

In addition to the acute inflammatory milieu at the time of AMI, it is important to recognize that the inflammatory effects of elevated TGs extend well beyond the acute setting. Hypertriglyceridemia is increasingly understood as a chronic, low‐grade pro‐inflammatory state that promotes endothelial dysfunction, oxidative stress, and macrophage foam cell formation over time—processes that cumulatively accelerate atherosclerotic plaque burden and vascular vulnerability. Chronically elevated TGs stimulate the production of pro‐inflammatory cytokines such as interleukin‐6 (IL‐6) and tumor necrosis factor‐alpha (TNF‐α), and facilitate the generation of small, dense LDL particles that are particularly susceptible to oxidative modification and subendothelial retention. These sustained pathophysiological mechanisms are not merely background noise; they have been shown to carry independent long‐term prognostic significance. The triglyceride‐glucose (TyG) index—a surrogate marker of insulin resistance closely related to TG metabolism—has been demonstrated to predict long‐term major adverse cardiovascular events (MACE) in patients with high cardiovascular risk, underscoring the chronic cardiovascular burden imposed by triglyceride‐related metabolic dysregulation [20]. Furthermore, in patients with heart failure—a population in whom chronic neurohormonal and inflammatory activation is already heightened—the TyG index has been shown to independently predict long‐term mortality and appropriate implantable cardiac defibrillator (ICD) therapy, highlighting that triglyceride‐associated inflammation retains its prognostic relevance even in advanced chronic cardiac disease [21]. Taken together, these findings suggest that AIP, as a triglyceride‐rich lipoprotein‐based index, may not only reflect the acute inflammatory state at the time of PCI but may also serve as a marker of chronic atherogenic and inflammatory burden—a dimension that future longitudinal studies should explicitly investigate.

In addition to the AIP, this study incorporated triglyceride levels and white blood cell count into a predictive model. The constructed nomogram achieved an AUC of 0.745 (95% CI: 0.711–0.789), and internal validation confirmed the model’s good predictive performance. Elevated triglyceride levels are closely associated with the formation of sdLDL. High plasma TGs promote cholesterol ester transfer protein (CETP)‐mediated lipid exchange, leading to the enrichment of LDL cores with TGs. Subsequent lipolysis of these TGs within LDL particles stimulates the generation of sdLDL [22]. Furthermore, multiple studies have reported a positive correlation between inflammatory biomarkers and adverse cardiovascular events [23]. As a direct reflection of the inflammatory process, an elevated white blood cell count indicates a persistent subclinical inflammatory state. This state contributes to vascular endothelial injury and atherosclerotic plaque destabilization, thereby increasing the risk of SF/NR events.

## 5. Conclusion

In summary, the AIP may be associated with the risk of SF/NR following emergency PCI in patients with AMI. Measurement of the AIP, particularly when combined with white blood cell count, could potentially assist in the early identification of high‐risk individuals. However, the modest discriminative performance of the nomogram (AUC = 0.745) and the minimal incremental gain over AIP alone suggest that the current model has limited added clinical utility beyond AIP measurement alone. These findings require confirmation in larger, prospective, multicenter cohorts that include comprehensive procedural data and external validation. Such studies are essential before AIP can be recommended for routine clinical risk stratification in this setting.

## 6. Limitations

This study has several limitations. First, including both AIP and its component TGs in the same model introduces mathematical redundancy, though a sensitivity analysis excluding TGs confirmed the robustness of AIP as a predictor. Second, the exclusion of patients on preadmission statins or antiplatelet agents may have introduced selection bias and limited generalizability. Third, key procedural variables such as ischemic time, thrombus burden, GP IIb/IIIa inhibitor use, and stent characteristics were not included, weakening causal inference. Fourth, the TIMI flow‐based definition of SF/NR is subjective, and more objective measures would have been preferable. Fifth, the nomogram showed only modest improvement over AIP alone, limiting its incremental value. Finally, the single‐center, retrospective design without external validation precludes definitive conclusions. Future prospective, multicenter studies are needed to confirm these findings.

## Author Contributions

Qinyu Sun was involved in writing–original draft and funding acquisition; Yifan Deng was involved in methodology; Weiyi Zhou and Zhoujun Huang were involved in data curation; Shenghu He was involved in validation; Jing Zhang and Wei Zhou were involved in writing–review and editing. Qinyu Sun and Yifan Deng contributed equally. All authors have participated sufficiently in the work and agreed to be accountable for all aspects of the work.

## Funding

This study was financially supported by the Postgraduate Research & Practice Innovation Program of Jiangsu Province (NO: SJCX25_2401).

## Disclosure

All authors read and approved the final manuscript.

## Ethics Statement

This study complied with the tenets of the Declaration of Helsinki. The study protocol has been approved by our hospital’s ethical review board (NO: 2025ky251).

## Conflicts of Interest

The authors declare no conflicts of interest.

## Supporting Information

Additional supporting information can be found online in the Supporting Information section.

## Supporting information


**Supporting Information 1** Supporting Information Figure 1: Calibration curve of the nomogram. The predicted probabilities showed good agreement with observed probabilities, with no significant deviation from the ideal diagonal line (Hosmer–Lemeshow test: *χ* ^2^ = 6.92, df = 8, *p* = 0.812), indicating satisfactory calibration.

**Supporting Information 2** Supporting Information Figure 2: Decision curve analysis (DCA) of the nomogram. The nomogram provided a positive net benefit across a wide range of threshold probabilities, suggesting favorable clinical utility for risk stratification.

## Data Availability

The data that support the findings of this study are available from the corresponding author upon reasonable request.

## References

[1] Zhu D. , Zhang X. , Fang Y. et al., Identification of a Lactylation-Related Gene Signature as the Novel Biomarkers for Early Diagnosis of Acute Myocardial Infarction, International Journal of Biological Macromolecules. (2024) 282, no. Pt 6, 10.1016/j.ijbiomac.2024.137431. 39521235

[2] Mignatti A. , Echarte-Morales J. , Sturla M. , and Latib A. , State of the Art of Primary PCI: Present and Future, Journal of Clinical Medicine. (2025) 14, no. 2, 10.3390/jcm14020653. PMC11765626 39860658

[3] Kumar R. , Shaikh A. H. , Mir A. et al., Prognosticating Short-Term Outcomes in Patients With STEMI-ACS and Intra-Procedural Slow-Flow/No-Reflow Phenomenon, Prognostizierung kurzfristiger Ereignisse bei Patienten mit ST-Hebung und Aakutem Koronarsyndrom mit Intraprozeduralem Slow-Flow-/No-Reflow-Phänomen. Herz. (August 2025) 51, no. 1, 56–63, 10.1007/s00059-025-05326-w. 40810822

[4] Yang Q. , Zhu X. , Zhang L. , and Luo F. , Dyslipidemia and Aging: The Non-Linear Association Between Atherogenic Index of Plasma (AIP) and Aging Acceleration, Cardiovascular Diabetology. (2025) 24, no. 1, 10.1186/s12933-025-02695-8. PMC12023499 40281579

[5] Liu Y. , Li W. , Zhou H. et al., Association Between Atherogenic Index of Plasma and New-Onset Stroke in a Population With Cardiovascular-Kidney-Metabolic Syndrome Stages 0–3: Insights From CHARLS, Cardiovascular Diabetology. (2025) 24, no. 1, 10.1186/s12933-025-02732-6. PMC12004660 40241126

[6] Mo D. , Zhang P. , Zhang M. , Dai H. , and Wang G. , Association Between the Atherogenic Index of Plasma and Incident Hypertension Across Different Blood Pressure States: A National Cohort Study, Cardiovascular Diabetology. (2025) 24, no. 1, 10.1186/s12933-025-02775-9. PMC12093804 40399995

[7] Afsin A. , Kaya H. , Suner A. et al., Plasma Atherogenic Indices are Independent Predictors of Slow Coronary Flow, BMC Cardiovascular Disorders. (December 2021) 21, no. 1, 10.1186/s12872-021-02432-5. PMC8686646 34930134

[8] Bolognese L. , Carrabba N. , Parodi G. et al., Impact of Microvascular Dysfunction on Left Ventricular Remodeling and Long-Term Clinical Outcome After Primary Coronary Angioplasty for Acute Myocardial Infarction, Circulation. (2004) 109, no. 9, 1121–1126, 10.1161/01.CIR.0000118496.44135.A7. 14967718

[9] Ndrepepa G. , Tiroch K. , Fusaro M. et al., 5-Year Prognostic Value of No-Reflow Phenomenon After Percutaneous Coronary Intervention in Patients With Acute Myocardial Infarction, Journal of the American College of Cardiology. (2010) 55, no. 21, 2383–2389, 10.1016/j.jacc.2009.12.054. 20488311

[10] Atwood J. , Management of Acute Coronary Syndrome, Emergency Medicine Clinics of North America. (November 2022) 40, no. 4, 693–706, 10.1016/j.emc.2022.06.008. 36396216

[11] Kumar D. , Ahmed I. , Bardooli F. et al., Techniques to Treat Slow-Flow/No-Reflow During Primary Percutaneous Coronary Intervention, Cardiovascular Revascularization Medicine. (2023) 47, 1–4, 10.1016/j.carrev.2022.09.014. 36266151

[12] Eitel I. , Saraei R. , Jurczyk D. et al., Glycoprotein IIb/IIIa Inhibitors in Acute Myocardial Infarction and Angiographic Microvascular Obstruction: the REVERSE-FLOW Trial, European Heart Journal. (2024) 45, no. 47, 5058–5067, 10.1093/eurheartj/ehae587. 39217605

[13] Niccoli G. , Burzotta F. , Galiuto L. , and Crea F. , Myocardial No-Reflow in Humans, Journal of the American College of Cardiology. (July 2009) 54, no. 4, 281–292, 10.1016/j.jacc.2009.03.054. 19608025

[14] Wang L. , Liu J. , Wang Y. et al., Atherogenic Index of Plasma is an Independent Predictor of No-Reflow in Patients With ST-Segment Elevation Myocardial Infarction Undergoing Primary Percutaneous Coronary Intervention, Journal of Atherosclerosis and Thrombosis. (2022) 29, no. 8, 1159–1170.

[15] Çınar T. , Hayıroğlu M. İ. , Tanboğa İ. H. et al., Atherogenic Index of Plasma Predicts No-Reflow Phenomenon in Patients With Acute Coronary Syndrome, Biomarkers in Medicine. (2021) 15, no. 14, 1237–1246.

[16] Shi Y. and Wen M. , Sex-Specific Differences in the Effect of the Atherogenic Index of Plasma on Prediabetes and Diabetes in the NHANES 2011-2018 Population, Cardiovascular Diabetology. (January 2023) 22, no. 1, 10.1186/s12933-023-01740-8. PMC9887826 36717829

[17] Balling M. , Nordestgaard B. G. , Langsted A. , Varbo A. , Kamstrup P. R. , and Afzal S. , The Association Between Small Dense Low-Density Lipoprotein Cholesterol and Peripheral Artery Disease: A Large-Scale Cohort Study and Meta-Analysis, European Journal of Preventive Cardiology. (2025) 10.1093/eurjpc/zwaf305. 40367137

[18] Chen Y. , Fu Y. , Wang S. et al., Clinical Significance of Neutrophil Gelatinase-Associated Lipocalin and sdLDL-C for Coronary Artery Disease in Patients With Type 2 Diabetes Mellitus Aged ≥ 65 Years, Cardiovascular Diabetology. (November 2022) 21, no. 1, 10.1186/s12933-022-01668-5. PMC9682485 36397150

[19] Liu H. , Liu K. , Pei L. et al., Atherogenic Index of Plasma Predicts Outcomes in Acute Ischemic Stroke, Frontiers in Neurology. (2021) 12, 10.3389/fneur.2021.741754. PMC8542679 34707558

[20] Hayıroğlu M. İ. , Çınar T. , Çiçek V. , Palice A. , Ayhan G. , and Tekkeşin A. İ. , The Triglyceride-Glucose Index Can Predict Long-Term Major Adverse Cardiovascular Events in Turkish Patients With High Cardiovascular Risk, Journal of Lipid and Atherosclerosis. (September 2022) 11, no. 3, 280–287, 10.12997/jla.2022.11.3.280. 36212749 PMC9515730

[21] Özcan K. S. , Hayıroğlu M. I. , and Çınar T. , Admission Triglyceride-Glucose Index is Predictor of Long-Term Mortality and Appropriate Implantable Cardiac Defibrillator Therapy in Patients With Heart Failure, Biomarkers in Medicine. (May 2023) 17, no. 10, 487–496, 10.2217/bmm-2023-0113. 37522225

[22] Jiang S. , Zhang F. , Yang H. et al., Estimated sdLDL-C as a Biomarker of Hepatic Steatosis Severity in MASLD: A Retrospective Study, BMC Gastroenterology. (March 2025) 25, no. 1, 10.1186/s12876-025-03759-5. PMC11907928 40082781

[23] Dugani S. B. , Moorthy M. V. , Li C. et al., Association of Lipid, Inflammatory, and Metabolic Biomarkers With Age at Onset for Incident Coronary Heart Disease in Women, JAMA Cardiology. (2021) 6, no. 4, 437–447, 10.1001/jamacardio.2020.7073. 33471027 PMC7818181

