# Supplementary material for: Association Between the Atherogenic Index of Plasma and the Occurrence of Slow Flow/No‐Reflow in Patients With Acute Myocardial Infarction Undergoing Emergency Percutaneous Coronary Intervention
Source: Cardiol Res Pract. 2026 Sep 12;2026:4736031. doi: 10.1155/crp/4736031 (PMC13570492; doi:10.1155/crp/4736031)
Supplement: Supplementary file 2 [file CRP-2026-4736031-s002.pdf]

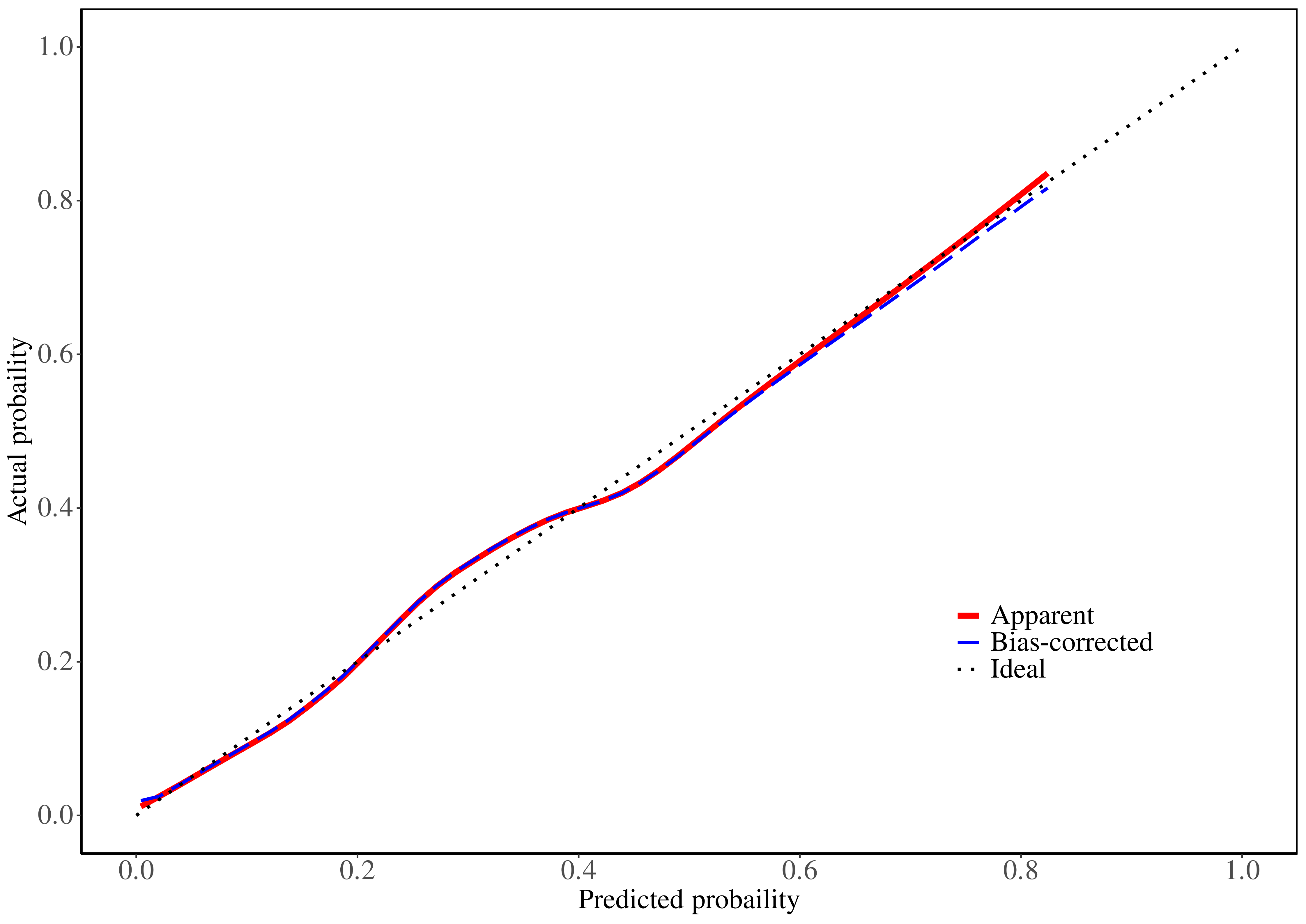
Actual probaility
1.0
0.8
0.6
0.4
0.2
0.0
0.0
0.2
0.4
0.6
0.8
1.0
Predicted probaility
Apparent
Bias-corrected
Ideal
